# Enzymatic and non-enzymatic oxidation of fibrillar collagen

**DOI:** 10.1080/13510002.2026.2682046

**Published:** 2026-06-11

**Authors:** Felipe Caliani Mathias-Netto, Nathalia Margarida Cantuária, Renato Simões Gaspar

**Affiliations:** a Department of Pharmacology, Faculty of Medical Sciences, University of Campinas (UNICAMP), Campinas, Brazil

**Keywords:** Collagen, oxidation, reactive oxygen species, post-translational modification, protein biophysics, oxidative stress, protein oxidation, crosslink

## Abstract

**Objectives:**

Collagen is a long-lived protein present in the extracellular matrix of force-bearing tissues. It has a unique amino acid composition of predominantly glycine, proline and hydroxyproline that repeats throughout its characteristic triple helical structure. In the extracellular space, collagen interact first by a non-enzymatic, entropy-driven process given their high hydrophobicity. Then, enzymes, such as lysyl oxidase (LOX), create covalent bonds (i.e. crosslinks) between triple helices, generating reactive oxygen species (ROS) as a byproduct. Moreover, it was recently discovered that collagen itself generates ROS upon stretching. Therefore, given the close proximity of ROS-generating sources, it seems plausible that collagen undergoes non-enzymatic oxidation.

**Methods:**

This review discusses collagen structure, mechanisms of crosslink formation and collagen oxidation.

**Results:**

Despite abundant data on the mechanisms of LOX-mediated collagen oxidation, there is sparse data on the effects of non-enzymatic oxidation on collagen chemical and biophysical properties, as well as its effects on cells and tissues.

**Discussion:**

The premise that collagen oxidation could lead to persistent damage is discussed in light of the immunogenicity and proteolysis induced by such modifications. Overall, data support that oxidative modifications in collagen should be further explored and could pose as a novel underlying mechanism in ageing and chronic diseases.

## Introduction

The extracellular matrix (ECM) is an essential component of the human body with a wide range of functions, such as cell differentiation [1], tissue strength and elasticity [2], while also providing anchoring points to the cells that compose tissues and organs [3]. To execute these functions, the ECM is formed by several proteins, such as collagens [4], fibronectin [5], laminins [6], elastin [7], among others. All these proteins contribute to tissue stability and elasticity, cell anchoring, tissue modelling and wound healing.

Among the proteins that compose the ECM, collagen is the most prominent, corresponding to around 30% of the total protein mass contained in the human body. There are almost 30 collagen types identified with different structures, functions and locations [4]. In this sense, collagens influence several physiological processes, such as cell differentiation and adhesion [8], while providing structural integrity and resistance to tissues such as tendons [9] and muscles [10], by acting on processes that involve power transfer between muscle fibres.

Given their various functions, collagens' complex genetic variations, assembly and structures have been studied in detail and extensively reviewed [11]. Collagens are large proteins, reaching sizes of up to 300 kilodaltons (kDa), with the majority of collagen fibre types being composed of a triple helix heterotrimer. Among the several types of collagens, the most predominant, and the focus of this review, is type I collagen (herein referred to as collagen), accounting for ~90% of the total collagen mass in the body. While collagen shares structural and functional similarities with other collagen types, such as types II and III, each type is implicated in different functions and locations. Given such heterogeneity, the way these proteins assemble and form crosslinks directly affects their structure and how adherent cells signal.

Such processes (both the formation of crosslink and cell signalling induced by collagen) intertwine with redox signalling mediated by reactive oxygen species (ROS). Although it is unclear whether the pro-oxidant dysfunctions present in chronic diseases affect the collagen structure per se, several associative data suggest that excessive oxidant generation affects the ECM and cell signalling [12,13]. Therefore, this review focuses on the biochemical aspects related to the enzymatic and non-enzymatic oxidation of collagen molecules. There are extensive data on lysyl oxidase (LOX)-mediated oxidation of collagen—however, there is sparse literature on non-enzymatic mechanisms. Since most previous reviews focused solely on crosslinks formed by glycation [14–16], we have compiled the literature on how ROS directly alter collagen structure and function and briefly discuss the glycation mechanism. The data herein suggest that collagen oxidation could be implicated in the pathophysiology of chronic non-communicable diseases, although mechanisms—chemical, biochemical and biophysical—remain sparse.

## Collagen crosslinking and chemical structure

Collagen chains are mainly composed of three amino acids: glycine (Gly), proline (P) and hydroxyproline (O). Together, these three make up almost 60% of the collagen chain [17]. Glycine, also called amino acetic acid, is positioned at the beginning of the triple amino acid sequence that repeats itself throughout the collagen chain (Gly-X-Y, in which X is usually P and Y is commonly O). Glycine-rich proteins are often flexible and malleable since glycine is the smallest essential amino acid, allowing sharp bends [18].

Due to its simple structure, glycine is a relatively simple molecule that, in its crystalline form, forms a double-layered crystal held together by hydrogen bonds [19]. This characteristic allows glycine to act as a connector between other amino acids of different polarities, and its small size is crucial for maintaining the triple helical structure of collagen. Its high predominance, comprising about a third (circa 35%) of total collagen amino acids, generates little steric hindrance, different from larger and more complex amino acids, thus allowing the molecule to fold into its characteristic shape. The placement of glycine in the collagen chain is also crucial for the stability and conformational structure of the collagen fibre [20,21].

The presence of the hydroxyproline residue is also vital for the correct folding of the helix. When located at the Y position in the polypeptide chain, the hydroxyl group in hydroxyproline promotes the formation of the protein backbone, which is crucial for helix stability and proper conformation [22]. While stabilising the protein structure through its electronic properties, O also plays a significant role in the hydration of the collagen molecule through its hydroxyl group, which acts as an anchoring point for water molecules absorbed inside the collagen chain. These water molecules create intermolecular bridges between the three chains that make up the protein, further enhancing its stability [23].

The remaining part of the collagen structure contains several other residues that contribute to fibre stability, flexibility and mechanical resistance. Residues such as serine, asparagine, alanine, valine, methionine, isoleucine, tyrosine, phenylalanine, histidine, lysine, arginine and leucine can also be found in lesser amounts [24]. Notably, the presence of the precursor of asparagine, aspartic acid, can increase collagen stability when present in the X position of the chain [25].

Lysine residues can promote the interaction between collagen and salt composites in a plane parallel to the triple helix, which increases the melting temperature of the fibre, thus improving its resistance to thermal degradation [26]. This is due to the action of lysyl oxidase (LOX), which converts lysine residues to allysine, an aldehyde able to form covalent cross-links between molecules, providing tensile strength to the collagen molecule [27]. Other amino acids, such as cysteine, which contain sulphur, are essential for the correct folding and formation process of the triple helix structure inside the endoplasmic reticulum (ER) (pro-collagen) [28]. Histidine also promotes fibre stability through binding to three other amino acids [29].

Beyond their structural role in mature collagen fibres, cysteine residues have a central role in an early oxidative process that occurs prior to the extracellular crosslinking during the development of fibres in the ER. During its intracellular maturing process, the procollagen molecule goes through the formation of disulphide bonds between cysteine residues in the C-propeptide domain, which are essential for the correct registration of the three α-chains that compose the mature fiber, forming the backbone of the triple helix structure from the C to the N terminal. This process, recently conceptualised as the cysteine code, establishes that the pattern of disulphide bond formation mediated by ER oxidoreductases, such as protein disulphide isomerase A1 (PDIA1), determines whether the triple helix is correctly folded or sent for degradation [30]. This highlights the central role of redox processes not only in the ECM but also inside the cell during the earlier stages of the procollagen molecule maturation in the ER. During this early stage of maturation, procollagen undergoes a series of changes catalysed by a complex enzymatic system composed of chaperones, including the collagen-specific chaperone Hsp47 and lysyl hydroxylase 2 (LH2) [31]. The disruption of the redox balance during the biosynthesis of procollagen, as observed in conditions of oxidative stress, can compromise procollagen folding by the enzymes present in the ER, depicting another mechanism by which unbalanced oxidative conditions may impact collagen structure and function.

**Figure 1.**
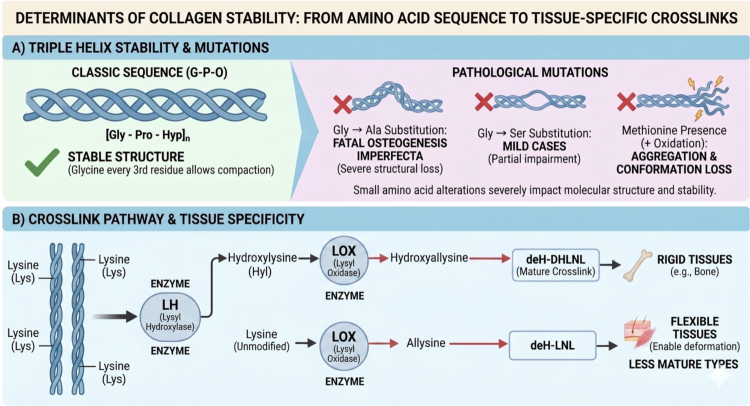
Collagen structure: from amino acids to crosslinks. Small substitutions in collagen amino acids are related to several diseases, such as osteogenesis imperfecta (A). In the crosslinking pathway (B), lysyl hydroxylase (LH) acts on lysine residues to form hydroxylysine (Hyl), which is subsequently oxidised by lysyl oxidase (LOX) into hydroxyallysine, leading to the formation of deH-DHLNL—a mature crosslink found predominantly in rigid tissues such as bone. In the absence of prior hydroxylation by LH, LOX acts directly on unmodified lysine to produce allysine, forming the less mature deH-LNL crosslink, which is associated with flexible tissues. The two pathways illustrate how the extent of LH-mediated hydroxylation before LOX activity determines crosslink maturity and tissue mechanical properties.

While modifications in specific amino acids can improve collagen stability and strength, others are associated with pathological changes. For instance, a substitution of glycine with alanine is related to fatal cases of osteogenesis imperfecta, while the substitution with serine leads to mild cases. The presence of methionine, an amino acid that is more susceptible to oxidation, can, on the other hand, induce the loss of protein structural conformation, forming aggregates [32]. These findings evidence how small amino acid residue alterations can severely impact molecular structure and stability, either by promoting stability or compromising it (Figure 1A).

In addition to the amino acids that confer properties of collagen monomers, the formation of fibrils and fibres depends on inter-molecular interactions that provide structural integrity to the ECM. Interactions between different collagen molecules, a phenomenon called collagen crosslinking, are mediated through enzymatic reactions and redox processes (Figure 1B) and are crucial for matrix cohesion [33].

Collagen crosslinking can be classified according to the amino acids involved in the bond, as reviewed by Piersma & Bank [34]. The formation of mature (or trivalent) collagen crosslinks depends on the lysyl oxidase (LOX) enzyme, separating the types of crosslinks according to the amino acids involved. Hydroxyallysine is a crucial step in the formation of mature collagen bonds. The formation of hydroxyallysine depends heavily on the previous hydroxylation of lysine by Lysyl Hydroxylase (LH), forming hydroxylysine, which is then oxidised into Dehydro-dyhydroxylisino-norleucine (deH-DHLNL) if both amino acids are hydroxylated, dehydro-lysine-norleucine (deH-LNL) if neither of them is hydroxylated, or dehydro-hydroxylysine-norleucine (deH-HLNL) if only one of them is hydroxylated by LH [34]. While deH-DHLNL is found in rigid tissues, the others are considered ‘less mature’ collagen crosslink types, such as HLNL and histidine-hydroxymerodesmosine (HHMD), which are more commonly found in flexible tissues [35], such as the skin and muscles, where such less rigid crosslinks enable tissue deformation without rupture (Figure 1B) [36].

## Enzymes involved in collagen crosslinking

**Figure 2.**
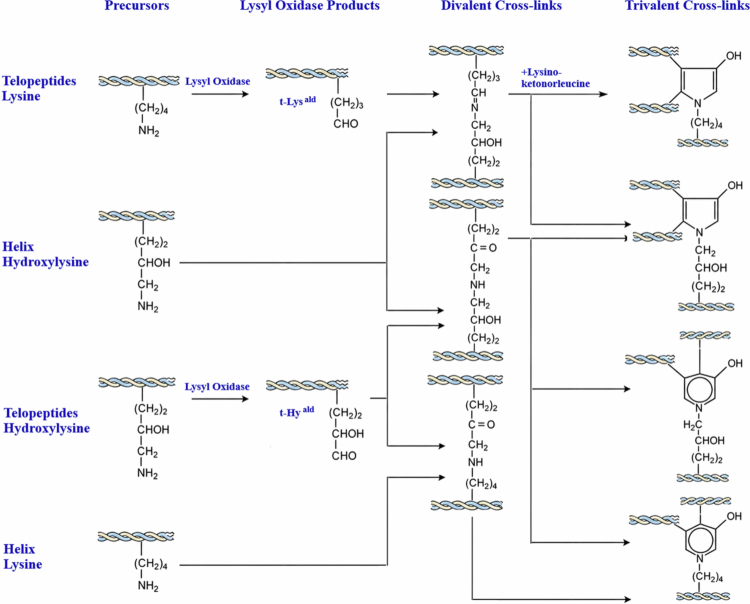
The crosslinking biosynthesis pathway is mediated by the lysyl oxidase (LOX) enzyme family. Lox enzymes catalyse the oxidative deamination of lysine residues (Lys) and hydroxylysine (Hyl) present at the telopeptides and helicoidal regions of collagen, generating allysine (t-Lysald) and hydroxylysine (t-Hyald), respectively, with the simultaneous release of NH3 and H_2_O_2_ as byproducts of this redox process. The aldehydes then make a spontaneous reaction with nearby amino residues, mainly constituted of Lys and Hyl, forming Schiff bases, generating immature divalent crosslinks (deH-HLNL) when both residues are hydroxylated, or deH-LNL, in the absence of previous hydroxylation. The subsequent maturing process occurs by the incorporation of a third residue, resulting in stable trivalent crosslinks. The hydroxylated pyrroles derived from Hyl and the pyridinamine and deoxypyridinoline derived from the Lys pathway provide fibrillar collagen with its definitive mechanical characteristics.

The primary source of collagen crosslinking is the Lysyl Oxidase enzyme family, which in mammals comprises four enzymes: lysyl oxidase (LOX) and four LOX-like enzymes, named LOXL-1 to 4, that act mainly on fibrillar collagens I, II, III, V and XI and elastin [37]. The LOX enzyme family is characterised by a catalytic carboxyl-terminal with different residues that determine the specificity of each enzyme. They are highly dependent on copper ions (Cu^2+^), which act as a structural factor and provide the electron flux essential for the enzymatic reactions to occur [38]. Upon release from the cell, collagen molecules undergo non-enzymatic binding, given their high hydrophobicity, forming small fibrils. Then, LOX enzymes act selectively on these recently formed fibrils, causing the deamination of lysine and hydroxylysine residues to form allysine and hydroxyallysine, respectively, enabling stable crosslinking between collagen telopeptides, which are essential for fibrillogenesis [26]. As a byproduct of this reaction, a significant amount of hydrogen peroxide (H_2_O_2_) is produced (Figure 2). Under physiological conditions, H_2_O_2_ released by reactions between collagen and LOX enzymes acts as a signalling molecule that modulates fibroblast gene expression, influencing other enzymes present in the ECM [39]. However, in pathological states where LOX is overexpressed, higher levels of H_2_O_2_ can lead to oxidative damage to matrix proteins and cells in the vicinity, as observed in heart disease [40].

One of the main mechanisms regulating LOX activity is tissue oxygen pressure. During hypoxia, hypoxia-inducible factor 1α (HIF-1α) promotes LOX expression by interacting with hypoxia response elements (HREs) [41], a crucial step in tissue recovery after injury, due to its role in fibrillogenesis, which takes part in wound healing [42]. After LOX protein synthesis, its signal peptide needs to be removed by bone morphogenetic protein 1 (BMP-1) via a reaction mediated by periostin, a secretory protein found in dense connective tissues. Together, they promote the correct cleavage of LOX enzymes and act as regulators of collagen crosslinking, as the amount of cleaved LOX limits collagen crosslinking [43].

Despite its predominant role, the LOX pathway is not the only enzymatic mechanism of collagen crosslink formation. During tissue repair, transglutaminases, particularly transglutaminase 2 (TG-2), can act as alternative mechanisms for collagen crosslinking [44], and this specific enzyme has also been documented to act in this process during the formation of fibrotic tissue. Unlike LOX, which relies on copper ions and oxygen to form aldehydes, TG enzymes function independently of oxygen and require calcium ions (Ca^2+^) for proper function. The bond formed by TG-2 is formed between lysine and glutamine, in which the glutamine carboxamide is bound to lysine [45]. The direct peptide bond forms a resistant, mechanically rigid connection between the two residues, a characteristic well observed in fibrotic tissues [44].

LOX differs from other ROS-producing enzymes, such as NADPH oxidases, because it does not require a dissociable cofactor. Instead, it establishes a permanent covalent bond using the lysyl tyrosylquinone cofactor (LTQ), which is derived from the crosslink between a tyrosine and a lysine in the LOX enzyme chain [46]. For LTQ to be formed, a copper (Cu) atom must be positioned near the two residues, catalysing the hydroxylation and oxidation of the lysine residue [47].

Following enzyme post-translational modifications (PTMs) that are essential for its correct functioning, LOX is secreted from the Golgi complex into the ECM, where, upon coming in contact with recently formed collagen fibrils, it reacts with lysine and hydroxylysine residues in an oxidoreduction reaction. During the reductive step of the process, LOX removes a proton (H^+^) from the lysine residue in the rate-limiting step of the reaction. After proton removal, LOX breaks the bond to the collagen residue, turning it into allysine in a step that leaves LTQ in a reduced state [48]. After the reduction of half of the reaction, the enzyme is oxidised by molecular oxygen, which receives electrons mediated by the copper ion at the active site. The reduced oxygen then removes the proton that was acquired during the reduction phase, forming hydrogen peroxide and ammonia (NH_3_), restoring the LTQ to its original state and restoring the enzyme to its original state, where it can take part in future reactions [49].

Despite sharing a common mechanism, different isoforms of the LOX enzyme have significant differences in their sequence, mainly in their N-terminal regions, which are crucial for determining how they interact with their ligands. These differences divide the LOX enzymes into two groups: the LOX and LOXL1 subfamilies and the LOXL-2 subfamily [37].

The first subfamily, composed of the main LOX enzyme and LOXL-1, is characterised by disordered N-terminal regions that are poorly conserved. These enzymes are secreted into the ECM and rely on post-translational modifications occurring outside of the cell [50]. Unlike LOX, LOXL-1 is specialised in the formation and maintenance of fibres present in tissues that undergo constant deformations, such as the skin, lungs and muscles. To form crosslinks, LOXL-1 relies on a cofactor, Fibulin-5, also called Developing Arteries and Neural Crest EGF-like (DANCE). LOXL-1 cannot bind directly to collagen substrates and relies on Fibulin-5 to act as an intermediate to form these bonds. DANCE binds to tropoelastin through its N-terminal domains, while it recruits LOXL-1, anchoring the protein and positioning its catalytic site near the lysine and hydroxylysine residues, where LOXL-1 will undergo the reactions described above to form the collagen crosslinks [51].

The second subfamily is constituted by LOX-L2, 3 and 4, with subtype 2 being the most predominant. This group of enzymes is characterised by the presence of four scavenger receptor cysteine-rich (SRCR) domains, which are conserved modules of protein interaction. The presence of these domains allows the enzyme to have its catalytic properties without the removal of the N-terminal domains [52]. LOXL-2 is found mainly in fibrotic tissues and tumours. Its presence is related to the induction of epithelial‒mesenchymal transition, which reduces the adhesiveness and polarity of epithelial cells, leading to invasive phenotypes. LOXL-2 achieves this by removing amines from specific lysine residues in a manner that increases the pathological stiffness of several tissues by the activation of fibroblasts [53].

LOXL-3 has a similar structure and functions to LOXL-2 but differs in its expression patterns. LOXL-3 is highly expressed during embryogenesis, contributing to craniofacial and cartilage formation, with mutations on the LOXL-3 being related to bone disorders, such as severe cleft palate and vertebral malformation [54]. LOXL-3 has high affinity for collagen types that are predominant on cartilages, such as collagen II and XI, with crosslink malfunction on those collagen types leading to the abovementioned disorders.

**Figure 3.**
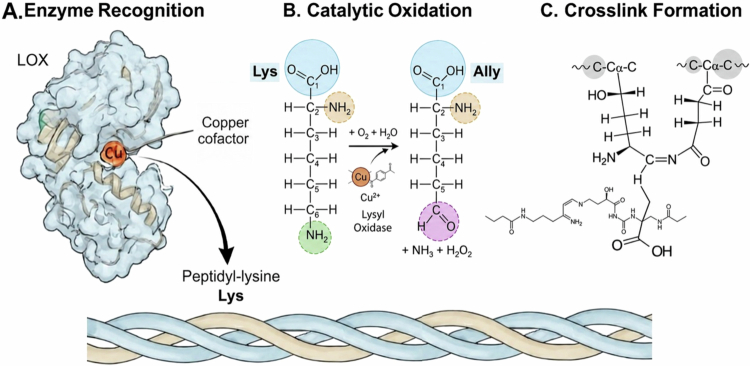
General mechanism of collagen crosslinking by lysyl oxidase. LOX recognises and binds to peptidyl-lysine (Lys) residues in collagen fibrils via its active site, which contains copper ions (Cu^2+^) (A), which are essential for catalysis (B). During catalytic oxidation, the copper ion acts as an electron transfer mechanism mediator, enabling the oxidative deamination of the amino group (−NH_2_) at the C6 position of the lysine. This reaction occurs in the presence of O_2_ and H_2_O, converting lysine into allysine (Ally), an aldehyde with a characteristic reactive group (C=O) also at C6. Ammonia (NH_3_) and H_2_O_2_ are released as products of this reaction. The Ally aldehyde goes through a spontaneous condensation with the amino group of a hydroxyallysine residue on a nearby collagen fibre, forming a Schiff base (C=N) that stabilises into a covalent intermolecular link that provides the collagen fibre with tensile strength (C).

LOXL-4 is the most recently found enzyme in the LOX family. Owing to its recent discovery, its function is still not as clear as that of other isoforms. The current understanding is that LOXL-4 is involved with tumorous tissues in a dual role, depending on the type of tumour, as LOXL-4 can act both as a tumour suppressor enzyme in tissues such as the bladder, where LOXL-4 expression has a cytostatic effect. Indeed, the silencing of the LOXL-4 gene was found in bladder cancer cells [55]. Nevertheless, in other types of cancers, such as head and neck squamous cell cancer [56] and breast cancer [57], LOXL-4 overexpression was related to uncontrolled cell proliferation and invasiveness. The structural and functional diversity of the LOX enzyme family is summarised in Table 1, while a more detailed illustration of its working mechanisms is provided in Figure 3.

**Table 1.** LOX family enzymes, co-factors, tissue expression and associated pathologies.

Enzyme	N-terminal Domain	Additional cofactor	Preferred tissue expression
LOX	Disordered, poorly conserved	None (LTQ + Cu^2+^)	Fibroblasts, ECM
LOXL-1	Disordered, poorly conserved	Fibulin-5 (DANCE)	Skin, lung and muscles
LOXL-2	Four SRCR domains	None (LTQ + Cu^2+^)	Fibrotic tissue, tumours
LOXL-3	Four SRCR domains	None (LTQ + Cu^2+^)	Cartilage (embryonic)
LOXL-4	Four SRCR domains	None (LTQ + Cu^2+^)	Context-dependent

The mammalian LOX family of enzymes shares a conserved catalytic carboxyl-terminal domain but differs in N-terminal organization, required cofactors, tissue distribution and associated diseases. LOX and LOXL-1 present disordered N-terminal regions and are dependent on post-translational modifications in the ECM, while LOXL-2, 3 and 4 contain four Scavenger Receptor Cysteine-Rich (SRCR) domains that enable catalytic activity without the removal of the N-terminal. All LOX enzymes require the presence of copper ions (Cu^2+^) and lysyl tyrosylquinone (LTQ) cofactor as catalysts. Fibulin-5 is listed as an additional cofactor of LOXL-1, as it is a necessary intermediate in the enzyme-substrate interactions. Associated pathologies are conditions in which aberrant expression of each enzyme isoform has been observed in previous studies.

## Enzymatic mechanisms of collagen oxidation

The collagen maturation process, composed of crosslink formation, is essential for ECM stability, being a complex and orchestrated process. During these maturation steps, collagen will go through several modifications, such as oxidation, mainly by the previously described action of the LOX enzyme family. These modifications not only alter the way that collagen interacts with nearby collagen molecules but also its solubility, going from a soluble immature fibre to a mature insoluble one. The process involving the LOX enzyme family occurs mainly on telopeptide domains from collagen types I and III, following the back-and-forth mechanism involving the oxidation of an amine to an aldehyde, as discussed above and previously described by Shah et al. [48] and Williamson & Kagan [49]. Recently, Akam-Baxter et al. [58] designed a sophisticated fluorescent probe to visualise allysine as a proxy for collagen oxidation. This same study found that, upon ischaemia–reperfusion injury, oxidised collagen accumulates in the hearts of mice and zebrafish, with remarkable differences between species—an effect likely due to the formation of mature vs. immature crosslinks. Interestingly, oxidised collagen was also found in the aorta of the mice, predominantly at the cross, where oscillatory shear stress occurs.

Bonds formed by the oxidation of lysine residues by LOX, forming allysine, tend to be more unstable due to higher susceptibility to pH and chemical cleavage, mainly because of the characteristics of the Schiff base. After allysine is formed, it reacts with another lysine, forming a dehydrolysine-norleucine (deH-LNL) crosslink. Its stabilisation relies on reduction processes that usually do not take place in an efficient way in the ECM, giving this crosslink its unstable characteristics [26]. Surprisingly, it was recently shown by Rennekamp et al. [59] that a specific trivalent (mature) bond, pyridinoline, functions as a sacrificial bond that ruptures upon stretching. Upon its rupture, the homolytic break generates ROS—i.e. mechanoradicals—that are readily scavenged by nearby amino acids, thus forming dihydroxy-phenylalanines (DOPA) [60].

The formation of hydroxyallysine differs from the formation of allysine in the step previous to LOX oxidation. Before that, the lysine residue is transformed into hydroxylysine by the Lysil Hydroxylase 2, adding a hydroxyl radical to the amino acid, which is then turned into hydroxyallysine by LOX. This additional step creates a much more stable basis for crosslink formation, allowing more stable crosslinks such as dehydro-hydroxylysine-norleucine (deH-HLNL), a crosslink found in abundance in more rigid tissues [26,48].

Therefore, crosslink formation is an enzyme-dependent process that relies on the oxidation of specific amino acids within collagen molecules. The formation of crosslinks by LOX generates ROS as a by-product. Likewise, the breakage of sacrificial bonds by force stress (e.g. stretching) also generates ROS (presumably hydrogen peroxide). The presence of the transition metal ions Fe^2+^ and Cu^2+^ near LOX and collagen itself, coupled with the ROS-generating processes, supports the hypothesis that non-enzymatic oxidation of collagen amino acids may also occur—although definitive proof *in vivo* is lacking.

## Non-enzymatic oxidation

Non-enzymatic oxidation is an oxidative process that occurs independently of the action of specific enzymes and results from the interaction of reactive species (RS) with target molecules. Proteins, such as collagen, owing to their abundance, localization and structural characteristics, are major targets of reactions involving RS. These reactions lead to post-translational modifications (PTMs), which may alter protein structure and compromise their biological function [61–63].

RS can be generated through different mechanisms, both intracellularly and extracellularly, and their production may be triggered by endogenous as well as exogenous sources. RS comprise reactive oxygen species (ROS), reactive nitrogen species (RNS), reactive sulfur species (RSS), reactive halogen species (RHS) and reactive carbonyl species (RCS) [61,62]. This section will focus on ROS, although other modifications will be discussed in less detail, given the lack of original work on the topic.

**Figure 4.**
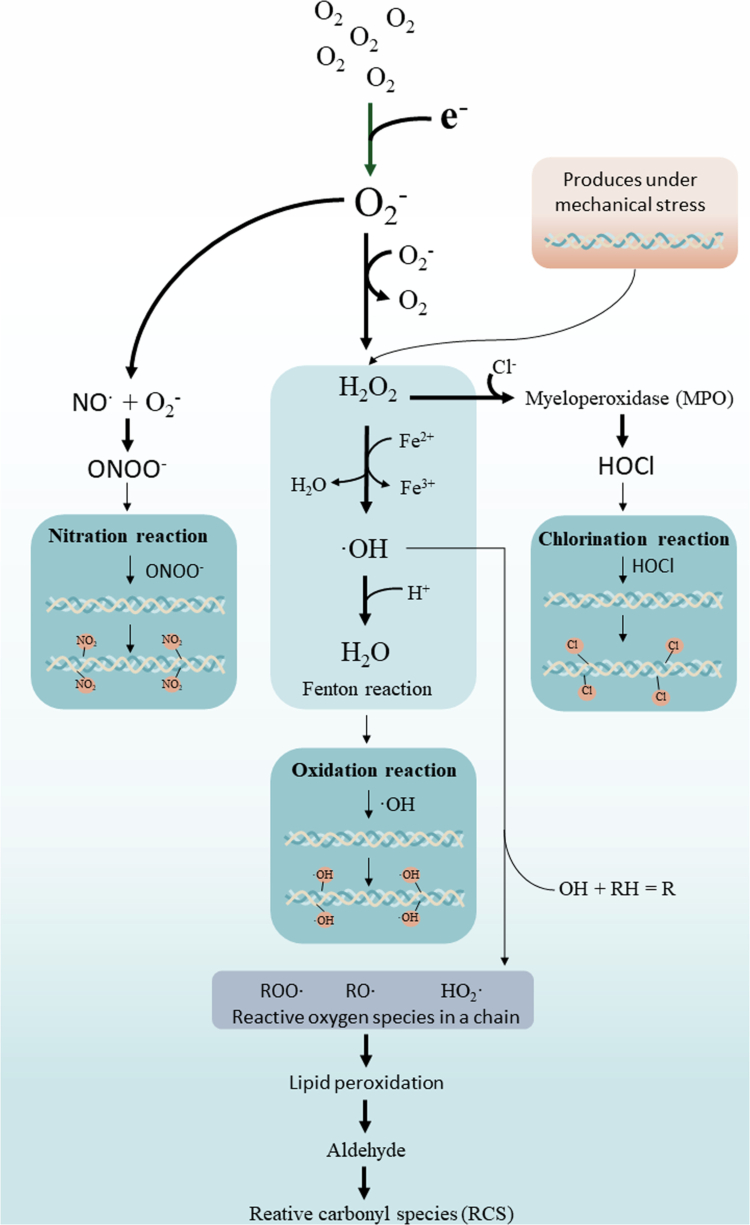
Generation of reactive species. The production of reactive species is initiated from molecular oxygen (O₂), whose electronic ground state is triplet oxygen (³O₂). Through the mitochondrial electron transport chain and/or the activity of NADPH oxidases or other enzymes, O₂ gains one electron, generating the superoxide anion radical (O₂.⁻). Superoxide is subsequently converted by superoxide dismutase (SOD) into hydrogen peroxide (H₂O₂). Hydrogen peroxide serves as a central precursor for the formation of reactive oxygen species (ROS) and reactive halogen species (RHS). Nitric oxide (NO·) reacts with superoxide (O₂⁻), giving rise to peroxynitrite (ONOO⁻), a reactive nitrogen species (RNS). ROS, RHS and RNS can oxidise the collagen present in the ECM, leading to structural modifications in the protein. It is also worth noting that collagen itself can generate H₂O₂. Other reactive species are generated as secondary products of these reactions, such as reactive sulphur species (RSS), which can arise from the interaction of ROS and RNS, as well as additional ROS formed through chain reactions, including lipid peroxidation processes.

ROS are classified into two groups: radical and non-radical species. Radical ROS include hydroxyl radical (·OH), superoxide anion (O₂.⁻), peroxyl radicals (ROO·), alkoxyl radicals (RO·) and the hydroperoxyl radical (HO₂·). These species contain one or more unpaired electrons, which makes them highly unstable and extremely reactive. Non-radical ROS include hydrogen peroxide (H₂O₂), singlet oxygen (¹O₂) and ozone (O₃). Although these species do not display unpaired electrons, they are strong oxidising agents and act as precursors for the formation of RS [62]. Figure 4 illustrates the generation of RS and highlights that most of them derive from molecular oxygen, while others, such as RSS, are generated as products of secondary reactions.

Some of these RS can react with virtually any biomolecule nearby, whereas others are more selective, reacting with more specific targets and in particular locations [64]. Oxidative PTMs derived from reactions with RS are diverse and depend on several factors, such as the target, the RS involved (in some cases, more than one species may be involved), and the biological response associated with the function of a specific protein.

The exacerbated production of ROS, together with an ineffective antioxidant system, leads to a state of oxidative imbalance known as oxidative stress. Although these molecules are essential in the organism due to their contribution to several biological processes, their excessive levels may cause undesirable reactions that culminate in PTMs of various biomolecules, contributing to the development and maintenance of chronic diseases, such as diabetes mellitus, cardiovascular diseases, cancer, and neurodegenerative diseases, as previously reviewed by several authors [62–65].

Found in the extracellular environment, GPx3 is an important antioxidant enzyme belonging to the glutathione peroxidase family that acts by catalysing the reduction of H_2_O_2_, hydroperoxides and lipid hydroperoxides, protecting different tissues from oxidative stress [66]. A study conducted with the objective of better understanding the functional aspects and production of GPx3 confirmed that GPx3 is essentially produced by the kidneys and that, in addition to being found circulating in blood plasma, it can also bind to specific basement membranes, such as those of the gastrointestinal tract, male reproductive tract, lungs and kidneys. The authors further emphasised that this binding is highly specific and occurs with specific cell types and that the binding takes place in regions where the regulation of the extracellular H_2_O_2_ concentration is necessary in targets that are more sensitive to oxidative stress damage [67]. Therefore, there is an antioxidant agent in basement membranes that is in the ECM of specific regions, even though it is still not clear how this interaction occurs.

The ECM, which is widely distributed throughout the organism, contains several components that are targets of RS [68]. Owing to its wide distribution, long half-life and protein structure, collagen is a relevant target for RS [68,69]. There are numerous studies in the literature addressing RS-induced PTMs of different proteins; however, few studies have explored PTMs in isolated collagen, making it an area that needs to be further developed. A significant portion of the available evidence and original studies date to the 1990s, when the idea of exploring these processes and relating them to the development and maintenance of chronic conditions emerged. Nevertheless, there is still much to be explored, particularly regarding the biophysical aspects of these modifications, and with the advancement of new technologies, it is possible to better understand how and where these modifications occur and how they are related to biological responses.

### Non-enzymatic oxidation of collagen

RS may react through different mechanisms, including hydrogen abstraction, electron abstraction and electron addition at electron-rich sites [64]. Protein modifications can be reversible, partially reversible, or irreversible. Reversible modifications play a signalling role, helping to indicate the state of oxidative stress, and often occur through the oxidation of the amino acid cysteine via processes such as oxidation, sulfenylation, S-nitrosylation and S-glutathionylation. On the other hand, an important irreversible modification that occurs in proteins is the addition of carbonyl groups (C=O) to side chains, known as carbonylation, which is a strong marker of oxidative stress. Some amino acids are more susceptible to this type of modification, such as Pro, His, Lys, Arg and Thr. Carbonylation contributes to the formation of cross-links in proteins that culminate in protein aggregation. Another form of irreversible reaction is nitration of the amino acid tyrosine, which is generated by reaction with ONOO^−^. These processes have been extensively reviewed by several authors [62–65]. The specific literature on how RS oxidises collagen molecules is discussed below.

Oxidising agents can be generated at both the intracellular and extracellular levels, and for oxidation reactions to occur, oxidising agents must be in proximity to the target molecule. Collagen is susceptible to oxidation by these agents. As discussed above, Zapp et al. [60] demonstrated that when subjected to mechanical stress conditions, collagen itself produces H₂O₂. Jang et al. [70] also demonstrated that when collagen activates platelets through binding the GPVI receptor, it leads to the production of H₂O₂. Later, it was found that GPVI activation is coupled with NADPH oxidase assembly, thus also generating superoxide [71]. Finally, the enzymatic oxidation of collagen by LOX generates H_2_O_2_ as a by-product [72]. Therefore, there are at least three oxidant-generating sources near collagen molecules: (1) collagen itself, (2) signalling cells and (3) LOX enzymes.

Some studies have observed modifications when collagen is exposed to superoxide, such as fragmentation of type I collagen, the role of extracellular superoxide dismutase [73] and collagen degradation under acidic conditions, with the release of 4-hydroxyproline [74,75]. There are also studies showing that histidinohydroxylysinonorleucine (HHL) and its precursor, the amino acid histidine (His), are targets of oxidation by singlet molecular oxygen (¹O₂), favouring the formation of cross-links between collagen fibres, which may contribute to fibre stiffness [76].

There are also a few studies investigating the interaction between H₂O₂ and collagen *in vitro*, despite H₂O₂ being one of the most abundant and widely distributed ROS. Liu et al. [77] evaluated how H₂O₂ contributes to the degradation of type I collagen extracted from tilapia skin in the context of gelatine production. Although the focus of the study was different, it provides relevant findings showing that the exposure of collagen to H₂O₂ promotes degradation and alters its organised structure. The authors demonstrated, through a mechanical resistance assay, that collagen fibres softened rapidly after incubation with H₂O₂. Scanning electron microscopy revealed that collagen fibre degradation increased with longer exposure time to H₂O₂ and that fibre bundles became looser. X-ray diffraction analyses showed that the characteristic triple-helical structure of collagen was damaged during the applied processes; however, Fourier transform infrared (FTIR) spectroscopy indicated that some helical structures were preserved even after treatment.

Nashchekina et al. [78] evaluated the effects of H₂O₂ exposure on the collagen structure and the responses of different cell types (adipogenic stromal cells (ASCs), MG-63 osteosarcoma cell line, A-431 epidermoid carcinoma, A-549 adenocarcinoma) to these changes. Scanning electron microscopy analysis showed a decrease in the collagen fibril diameter as the H₂O₂ concentration increased. The authors also assessed the water contact angle, as this property can influence protein adsorption and cell adhesion, and observed increased hydrophilicity of collagen fibres. This effect was attributed to the possible formation of new hydroxyl groups generated through collagen oxidation by H₂O₂. FTIR analysis revealed differences between the spectra of the control and oxidised collagen, including shifts in specific band peaks. Based on these findings, the authors suggested that changes in collagen structural organisation may have occurred, although intermolecular collagen bonds appeared to remain intact. The effects of this oxidation on several cell lines were evaluated. Oxidised collagen increased the adhesion of two of the tested cell types (ASCs and A-431), with a greater effect observed in A-431 cells. In contrast, increasing H₂O₂ concentrations led to a decrease in the number of adhered MG-63 cells. In a cell viability assay, one cell type (A-431) showed increased viability when exposed to oxidised collagen. These findings are highly relevant, as they demonstrate how non-enzymatic H₂O₂-mediated oxidation can induce structural changes in collagen and how these changes influence cellular responses.

Both O₂⁻ and H₂O₂ can lead to the production of ·OH through the Fenton and Haber–Weiss reactions, respectively, which are catalysed by iron or copper ions [79]. The hydroxyl radical is highly reactive and is considered the most reactive of all ROS, being capable of oxidising virtually any nearby protein. It also has a very short half-life (10⁻⁹ seconds), which makes its quantification and in vivo detection challenging [80]. Therefore, evidence of the presence of H₂O₂ near collagen favours the formation of ·OH through the Fenton reaction, using iron and copper ions available at the site. The presence of ·OH near collagen may lead to oxidation reactions, as previously described in the literature, culminating in collagen oxidation by a one-electron oxidising agent. It is worth noting that there are some estimates regarding the rate constant of ·OH reaction with collagen, which occurs practically at the diffusion limit (4 × 10⁸–10¹⁰ mol⁻¹.s⁻¹, pH 7.0) [63].

It is important to emphasise that ascorbic acid, an important antioxidant, is also capable of favouring the Fenton or Fenton-like reaction through the reduction of Fe^3+^ and Cu^2+^ ions, resulting in the propagation of the reaction, contributing to a faster reaction rate and consequently increasing the amount of ·OH generated [81–83]. In addition, ascorbic acid also acts as a cofactor for several enzymatic reactions, especially those involved in collagen maturation, such as prolyl-3-hydroxylase (P3H), prolyl-4-hydroxylase (P4H) and lysyl hydroxylase (LH) [84,85]. Altogether, evidence shows that there is an environment highly favourable for ·OH formation in close proximity to collagen.

Xiao; Cai and Liu, (2007) [86], demonstrated that when type I collagen is exposed to ·OH, structural modifications occur in the protein, as identified by FTIR spectroscopy. The authors observed that when collagen is in the presence of ·OH, there is a gradual decrease in the residual carbonyl group (C=O), as it would be participating in the reaction with ·OH. The authors discussed that the (C=O) group indicates the secondary structure of polyproline II based on findings from other studies [87] and that, due to collagen presenting several residual (C=O) groups, which are more exposed to the external environment, they become easy targets for reacting with ·OH. The authors also demonstrated through in vitro assays that collagen can eliminate ·OH from the medium, proposing that the protein could act as a scavenger of ·OH. This is in line with more recent works by Prof Frauke Grater's laboratory, as discussed above.

Another study [88], conducted with type II collagen oxidised by ·OH, identified structural alterations through UV absorption spectroscopy and circular dichroism. Oxidised collagen showed increased absorbance when compared to the non-oxidized condition. In circular dichroism analysis, a change in the negative signal was observed, indicating partial unfolding of the collagen and an alteration in the secondary structure. The study also demonstrated that collagen modified by ·OH exhibits increased immunogenicity and arthritogenicity.

It should be noted that PTMs might interfere with the proteolytic degradation of collagen. Some modifications, such as oxidative reactions, may increase protein clearance, as reviewed previously [89], whereas others, such as glycation, may reduce clearance [90,91]. However, evidence regarding collagen degradation following these PTMs remains limited, as few studies have explored this topic.

**Figure 5.**
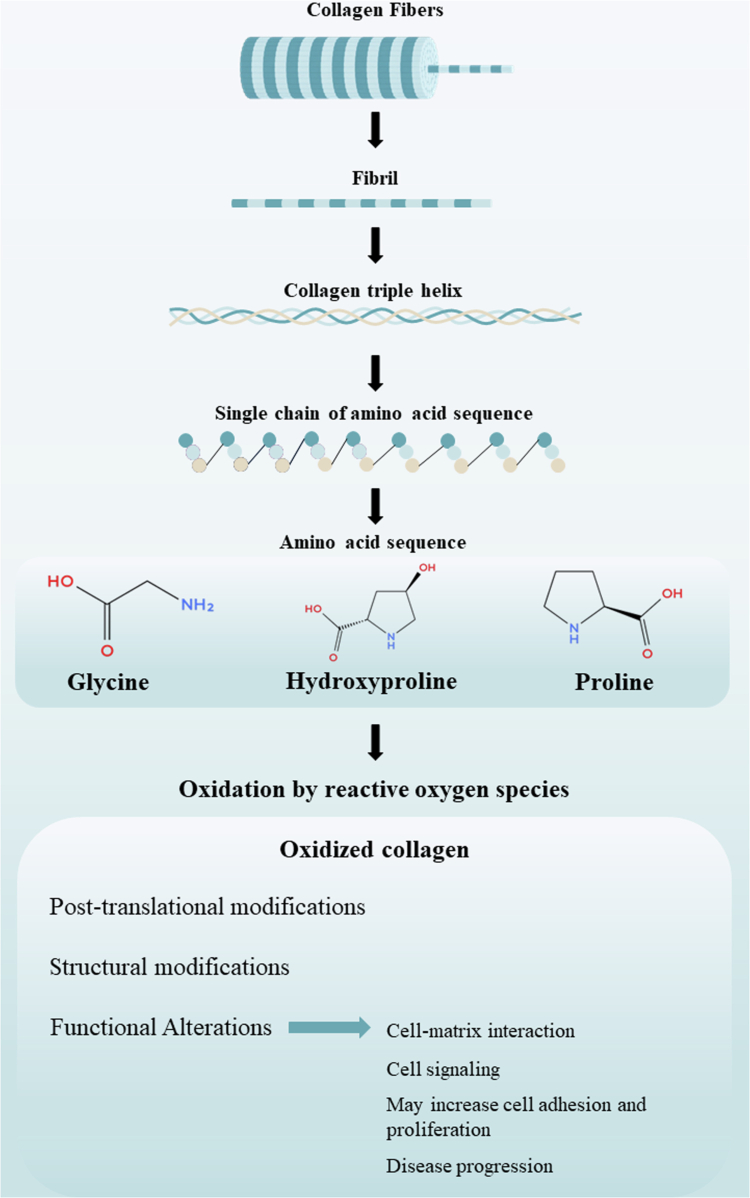
Collagen oxidation and functional effects. Collagen fibres are composed of multiple smaller fibrils. These fibrils are formed by a triple helical structure, and each helix consists of a single amino acid chain, with the amino acid sequence representing the primary structure. Three main amino acids constitute collagen: glycine, proline and hydroxyproline. The oxidation of collagen by ROS leads to post-translational modifications of the protein and structural alterations. These modifications may result in functional changes, interfering with cellular responses and various biological activities. The image does not depict specific targets or post-translational modifications, as these vary depending on the type of ROS involved. Moreover, there is insufficient evidence to support the notion that oxidation by a single ROS occurs at only one target or results in a single PTM. For this reason, the authors did not specify these details. However, the image illustrates the collagen structure and highlights potential targets and possible consequences of oxidative reactions involving ROS. The molecular structures were designed and optimized in 3D using MolView (http://molview.org/).

Considering the data reviewed herein, there is evidence that collagen reacts with ROS and that these culminate in PTMs (Figure 5). These PTMs cause important changes in protein structure and might interfere with collagen degradation, potentially leading either to the accumulation of modified collagen or to enhanced degradation, which could trigger an exacerbated compensatory synthesis. Given the long half-life of intact collagen and the data regarding its non-enzymatic oxidation, one could hypothesise that collagen oxidative PTMs could be associated with the development and progression of chronic diseases. The investigation of such a hypothesis might contribute to a better understanding of the underlying mechanisms and, consequently, to the identification of new pharmacological targets. Table 2 summarises the findings of previous studies on the oxidation, nitration and halogenation of collagen.

**Table 2.** Summary of the main outcomes of previous studies on the oxidation, nitration and halogenation of collagen.

	Reactive Species	Type of collagen	Outcome	Ref.
Oxidation reactions	O₂.⁻	Type I	Fragmentation of collagen [73] and collagen degradation [74,75]	[73,74,75]
	¹O₂	–	Formation of cross-links between collagen fibres [76]	[76]
	H₂O₂	Type I collagen from tilapia skin	Collagen degradation and alteration of the organised structure when exposed to H₂O₂	[77]
		Type I collagen from rat tail tendons	Decrease in collagen fibril diameter with increasing H₂O₂ concentration; increased hydrophilicity of collagen fibres; differences between the FTIR spectra of control and oxidized collagen; increased cell adhesion and viability	[78]
	OH	Type I collagen	Structural modifications in collagen identified by FTIR spectroscopy; gradual decrease in the residual carbonyl group (C=O); results show that collagen can act as an ·OH scavenger	[86]
		Type II collagen	In UV absorption spectroscopy, oxidised collagen showed higher absorbance; analysis by circular dichroism showed a change in the negative sign, suggesting an alteration in the secondary structure. Oxidized collagen also showed greater immunogenicity and arthritogenicity	[88]
Nitration reactions	ONOO⁻	Type II collagen	The modified collagen increased the inflammatory response of chondrogenic cells	[92]
Halogenation reactions	·OH, HClO, ONOO⁻	Type II collagen	Collagen fragmentation was observed when it was treated with the hydroxyl radical, hypochlorous acid, or peroxynitrite	[93]
	HOCl/OCl⁻	Type II collagen	The reaction with HOCl/OCl⁻ caused modifications in the amino acid side chains and secondary structure of collagen, as well as functional alterations, increasing immunogenicity and pathogenicity	[94]
	HOCl/OCl⁻ species, N-chloroamines and Cl₂,	Type II collagen	Oxidation of pyridinoline crosslinks	[95]
	HOCl/OCl⁻ species, N-chloroamines	Type II collagen	Exposure of collagen to HOCl caused protein fragmentation and prevented its gelation, while exposure to N-chloramines caused little fragmentation but an increase in degradation mediated by proteolytic activity	[96]

## Collagen and reactive nitrogen species and reactive halogen species

Whiteman et al. [92] evaluated the exposure of type II collagen to ONOO⁻ to understand how these modifications interfere with the development of inflammatory joint diseases such as osteoarthritis and rheumatoid arthritis. When compared with control collagen in chondrogenic cell cultures, ONOO⁻-modified collagen led to a greater inflammatory response, activating p38 MAPK and ERK pathways and the transcription factor NF-κB, resulting in increased production of NO and prostaglandin E2 (PGE-2). The authors discussed that exposure of collagen to this oxidizing agent not only causes modifications in protein structure but also contributes to different biological responses, favouring the inflammatory process under the evaluated conditions.

Another study also sought to evaluate whether type II collagen modified by different oxidizing species, such as the hydroxyl radical, hypochlorous acid and peroxynitrite, could lead to the production of neoantigenic epitopes in the context of rheumatoid arthritis [93]. The authors also evaluated the effects of glycation and observed differences in mobility between native collagen and glycated collagen, as well as differences in fluorescence. Collagen fragmentation was observed when it was treated with the hydroxyl radical, hypochlorous acid, or peroxynitrite. There is a wide distribution of oxidizing agents in inflamed joints, and according to the results of this study, modified collagen can act as an autoantigen in rheumatoid arthritis, contributing to disease chronicity and stimulating the immune system response against these autoantigens [93].

Olszowski et al. [94] demonstrated that type II collagen can undergo modifications when exposed to hypochlorite (HOCl/OCl⁻), which is produced by neutrophils by myeloperoxidases. The results of this study show that HOCl/OCl⁻ leads to modifications in amino acid side chains and in the secondary structure of collagen. Chlorination of the protein can alter several properties beyond its structure, such as immunogenicity and pathogenicity, which were related to rheumatoid arthritis.

Interestingly, a previous study used HOCl/OCl⁻ species, N-chloroamines and Cl₂ and demonstrated that these species can oxidise pyridinoline cross-links found in type II collagen present in articular cartilage [95]. These are the same crosslinks described by Rennekamp et al. [59] as those containing sacrificial bonds. Thus, one could speculate that oxidative PTMs of pyridinoline cross-links could either strengthen or weaken the collagen structure, although there is no study assessing this as far as we are aware.

Davies; Horwitz and Davies [96] evaluated the actions of HOCl and N-chloroamines on exposed collagen to assess how these reactions interfere with the collagen structure and its susceptibility to degradation by proteolytic enzymes. They used HOCl concentrations close to physiological levels and observed that, upon reacting with collagen, HOCl caused protein fragmentation and prevented its gelation. When collagen was exposed to N-chloroamines, the researchers observed little fragmentation; however, there was an increase in degradation mediated by proteolytic activity. Thus, it seems plausible that some PTMs may induce increased collagen proteolysis and may not accumulate over time.

## Other modifications—collagen glycation and glutathionylation

In addition to enzymatic and non-enzymatic oxidation processes, collagen is also susceptible to glycation. Protein carbonylation is a highly important process in the generation of irreversible PTMs. In addition to being introduced through reactions with RS, carbonyl groups can also be added through the reaction of proteins with products generated during lipid peroxidation and sugar oxidation processes [61].

During lipid peroxidation, in which oxidative reactions occur with polyunsaturated fatty acids, several products, such as lipid hydroperoxides, are released. These lipid peroxidation products are decomposed into different aldehydic compounds that exhibit high reactivity, such as 4-hydroxy-2-nonenal (HNE), 4-oxo-2-nonenal (ONE), acrolein and malondialdehyde (MDA). These final compounds tend to react with proteins, leading to the addition of carbonyl groups [97,98].

Furthermore, sugars such as ribose and glucose can react directly with the collagen molecule, specifically with the amino group residues of lysine and arginine, undergoing reduction and creating advanced glycation end-products (AGEs) [99]. Glyoxal (GO) can be generated as a product of both lipid peroxidation and the oxidation of reducing sugars. The main pathway to produce methylglyoxal (MGO) is glycolysis, which is associated with hyperglycaemic conditions; MGO can also be generated through the oxidation of reducing sugars [97,100].

Protein glycation and AGEs are closely related to the development of several pathophysiological conditions, particularly those associated with ageing, as they involve long-lived proteins, such as collagen [101]. AGEs that bind collagen can trigger structural alterations in proteins, such as fibre stiffening with reduced elasticity [99,102,103], and consequently contribute to various biological effects, such as hypertension and endothelial dysfunction. AGEs can activate the receptor for advanced glycation end products (RAGE), which is found in several cell types, especially in the lungs, thereby contributing to an exacerbated inflammatory response. A previous study demonstrated the effects of AGEs on the development of renal fibrosis in ageing, showing a decrease in renal fibrosis in old mice exposed to aminoguanidine, an AGE ‘inhibitor’ [104]. This process leads to increased ROS production. In this context, long-lived proteins, such as collagen, that have undergone glycation may activate RAGE receptors, leading to a sustained detrimental response.

Nowotny et al. [91] also investigated the effects of MGO-glycated collagen, showing that glycation of ECM proteins may be related to skin ageing and other chronic conditions, such as diabetes. The authors observed that modified collagen increased ROS production by fibroblasts, and this increase induced a condition of chronic stress within cells, which consequently led to cellular apoptosis. Therefore, glycation of collagen or other ECM proteins can generate pathological responses. A more extensive discussion of the effects of collagen glycation can be found in previous reviews [14–16].

In addition to the processes and reactions described above, another post-translational modification that occurs with collagen is glutathionylation. This reaction occurs through the conjugation of the tripeptide glutathione (GSH) with cysteine residues present in collagen. Druso et al. [105]. identified through immunoprecipitation an increase in collagen S-glutathionylation in lysed lung samples from patients with idiopathic pulmonary fibrosis (IPF). The processing of collagen 1 in the ER is regulated by different proteins that contribute to different stages, and the authors highlighted three proteins involved in redox regulation: PDIA3 (protein disulphide isomerase A3), ERO1A (endoplasmic reticulum oxidoreductase 1 alpha) and PRDX4 (peroxiredoxin 4). In IPF samples, an increase in PDIA3 was found, as well as an increase in the expression of ERO1A and PRDX4. The authors suggest that this increase occurs due to oxidative imbalance in the ER, which favours an oxidative environment and creates ideal conditions for collagen glutathionylation to occur. The authors also demonstrated that collagen S-glutathionylation increases secretion by fibroblasts, as well as increasing the proliferation of these cells, suggesting that this could be responsible for worsening the IPF condition. The role of the enzyme glutaredoxin (GLRX), which is responsible for deglutathionylation, was identified, as its suppression resulted in increased glutathionylated collagen. It was also observed that glutathionylated collagen is more resistant to degradation by collagenase [105].

## Conclusion and future perspectives

Collagen is a long-lived protein contained in the ECM that is fundamental to cell function in health and disease. It is mainly composed of glycine, proline and hydroxyproline, which confer the triple helical structure of collagen. In the extracellular space, LOX and other enzymes oxidise collagen, creating crosslinks that form fibrils, fibres and bundles. Much is known about the mechanisms of LOX-dependent oxidation and the chemical pathways upon which crosslinks are formed. However, little is known about how non-enzymatic oxidation and possibly other PTMs affect collagen. Recent data suggest that collagen itself is an ROS-generating source [60], while it has historically been known that ROS are a byproduct of LOX-catalysed reactions. Cellular signalling induced by collagens, such as the one mediated by the platelet receptor GPVI, also generates ROS. Therefore, it is plausible to hypothesise that collagen can undergo non-enzymatic oxidation, and that such PTMs could affect the development of chronic diseases, considering the half-life of collagen.

Future research should investigate how different oxidants modify the collagen structure and whether this leads to cellular dysfunction. It is important to identify, for instance, if oxidative PTMs affect many of the biophysical properties of collagen, such as its stiffness or piezoelectric properties. These could be studied using atomic force microscopy (AFM). It is also possible that oxidative PTMs may affect the entropic organization of tropocollagen in the extracellular milieu. Recent advances, such as high-speed AFM, may help in the study of such intricate phenomenon. Of note, it is possible that, upon oxidation, collagen may bind to different receptors than those bound by intact collagen, in a similar fashion that occurs with low-density lipoprotein (LDL) and oxidised LDL. Therefore, advanced molecular biology tools are needed to identify receptors for oxidised collagen. In addition, novel tools, such as the fluorescent probe against oxidised collagen developed by Akam-Baxter et al. [58], are necessary to track and identify specific PTMs in collagen induced by oxidants. This would help establish whether the accumulation of oxidised collagen in diseases is indeed an underlying mechanism or perhaps just an associative finding. Overall, further investigation in this perhaps overlooked field should yield novel perspectives into the old hypothesis that modifications in long-lived proteins can lead to long-lasting damage.
